# Learning results of GP trainers in a blended learning course on EBM: a cohort study

**DOI:** 10.1186/s12909-015-0386-2

**Published:** 2015-06-12

**Authors:** Ellen te Pas, Margreet Wieringa–de Waard, Wouter de Ruijter, Nynke van Dijk

**Affiliations:** 1 Department of General Practice/Family Medicine, Academic Medical Center, University of Amsterdam, location L0-310 Meibergdreef 9, Amsterdam, 1105 AZ The Netherlands; 2 Department of Public Health and Primary Care, Leiden University Medical Center, Leiden, The Netherlands

## Abstract

**Background:**

General practitioners (GPs) experience barriers to the use of evidence-based medicine (EBM) related to a negative attitude and to insufficient knowledge and skills. We therefore designed a blended learning intervention to develop the competence of GP trainers in EBM. This study investigated the effectiveness of this intervention in increasing the trainers’ EBM competencies (i.e. knowledge, skills, attitude and behaviour).

**Methods:**

In total 129 GP trainers participated in the blended learning course on EBM consisting of four 3-h face-to-face meetings and an intensive preparatory e-course before each meeting over a 12-month period. The primary outcomes were changes in knowledge and skills (Fresno test), changes in attitude (McColl test) and intentions to change behaviour. Secondary outcomes were changes in self-rated knowledge, skills and attitude, and the relation between personal characteristics and changes in knowledge, skills and attitude. Data were collected before the start of the intervention (T0), at the end of the last day of the intervention (T1) and four months after the end of the intervention (T2).

**Results:**

The mean changes in scores on the Fresno test were ∆T1-T0 = 40.8 (SD ±36.7, *p* < .001) and ∆T2-T0 = 20.8 (±39.9, *p* < .001). The mean changes in scores on the McColl test were ∆T1-T0 = 2.2 (SD ±12.8, p = .16) and ∆T2-T0 = -.87 (±10.0, p = .49). Of the GP trainers, 16.7 % fulfilled their intentions to change in behaviour, 47.6 % partly fulfilled them and 35.7 % did not fulfil them at all. Female trainers scored significantly higher on the Fresno test after the intervention compared to male trainers. There was a weak positive correlation between self-rated knowledge and the scores on the Fresno test. A moderate correlation was found between the overall score on the McColl test and self-rated attitude.

**Conclusion:**

An intensive blended learning course on EBM for GP trainers induces an increase in knowledge and skills that, although decreased, remains after four months. Attitude and behaviour towards EBM show no differences before and after the intervention, although GPs’ intention to use EBM more often in their practice is present.

## Background

General practitioners (GPs) experience several barriers to the use of evidence-based medicine (EBM) [1] related to a negative attitude towards the evidence itself and to the use of EBM in their clinical practice, as well as to insufficient knowledge and skills. They also experience barriers related to their practice and patient population [2], such as a lack of time, patient-related factors and a lack of available evidence. All these barriers [3] are also experienced by GP trainers despite the additional education they received to become trainers. Because GP trainers function as important role models for GP trainees [4, 5], modelling adequate EBM behaviour requires additional education to enhance their EBM competency (i.e. knowledge, skills, attitude and behaviour) [6].

GP specialty training in the Netherlands has a duration of three years. In the first and third years, the trainee works at the practice of a GP trainer. This GP trainer is also the daily supervisor of the trainee. In the second year, the trainee works for periods of three or six months in various clinical settings, such as a psychiatric care hospital or institute, an emergency department or a nursing home. New GP trainers (all of whom have at least five years of clinical experience as GP) follow a four-year training programme that mainly focuses on didactic competencies. EBM as a separate subject is currently not part of this training programme. After these four years, a GP trainer is required to follow eight days (48 h) of refresher courses each year at the training institute.

In today’s healthcare, EBM is considered essential for providing high quality patient care [7, 8]. Practising EBM requires the integration of clinical expertise, patient values and the best available evidence into the decision-making process for patient care [9]. Integrating these three elements into daily practice requires knowledge about and skills in EBM as well as a positive attitude [7, 9].

Fishbein and Ajzen’s [10] theory of reasoned action states that attitude and subjective norms (the influence of others in one’s social environment) are predictors of behaviour. Following this theory, to actually enhance the practice of EBM requires a change in attitude that will result in willingness to change behaviour.

Using education to change the EBM behaviour of GP-trainers thus requires a specified training program. These days, continuing medical education (CME) offers not only face-to-face (F2F) courses but also e-learning (e-courses) and, as the latest development, blended learning courses. E-courses are becoming more popular because of the didactical advantages they offer, such as the possibility to adapt them to individual learning styles [11] and paces, as well as the possibility to repeat courses [12, 13]. The logistic benefits, such as learning at any convenient time and place, are also seen as important advantages of e-learning in the context of CME [14–16]. A study by Kok and colleagues [17] among physicians in a non-hospital based medical specialty concluded that multiple educational methods should be used to change a physician’s behaviour.

Another study among occupational physicians [18] concluded that e-learning alone will increase EBM knowledge but it is not effective in increasing EBM skills and changing EBM behaviour. They recommended a blended learning course. Blended learning includes ‘different learning or instructional methods, different delivery methods, different scheduling and different levels of guidance’ [19]. These learning environments have both didactical and logistic advantages, while also offering learners interaction with peers on complex issues related to behaviour and professional role, and bridging the gap between theory and practice [12, 13, 20]. Blended learning also allows practice to be incorporated more fully into the learning pathway by including practice-based assignments [21].

Ilic and Maloney [22] performed a systematic review including EBM education in several kinds of educational modes for undergraduate and postgraduate medical students. Although they suggested that the findings of their review ‘provides educators with the option of adopting a flexible curriculum in EBM’, in their conclusion they stated that ‘it is not possible to determine which kind of intervention is most effective at increasing EBM competency at this point in time’ and that further studies are required. In a systematic review by Coomarasamy and Khan [23] they stated that EBM teaching should be incorporated into clinical teaching.

On the basis of the above considerations, we designed a blended learning intervention to enhance the EBM competency of GP trainers. In earlier studies, internet-based learning is compared with no educational intervention and/or traditional methods [24], and shown to be as effective as traditional methods. The primary aim of the present study therefore was to assess the magnitude in which this intervention increases GP trainers’ EBM competency. The secondary aims were to explore the relation between formal assessments as outcome measures and self-reported outcomes, and to determine whether specific characteristics of trainers are related to the outcomes of the intervention. We have designed this study as a cohort study as this design suited our objectives well.

## Methods

### Participants

122 of the 170 GP trainers at the Academic Medical Center, University of Amsterdam (AMC–UvA), and a self-selected group of 7 GP trainers at Leiden University Medical Center (LUMC) participated in the study. All the trainers were informed that participation in the study was voluntary and gave their informed consent. Ethical approval for this study was obtained from the Ethical Review Board of the NVMO (Netherlands Association for Medical Education).

### Setting

The use of clinical guidelines is common in Dutch GP practices; 100 guidelines developed by the Dutch College of General Practitioners (NHG) are currently available [25] to facilitate the use of EBM in general practice.

### Intervention

In 2012, the GP trainers at the AMC–UvA followed a blended learning course on EBM. The course consisted of four 3-h F2F meetings and a 12-h e-course divided into four 3-h modules spread over 12 months. In preparation for each meeting, trainers were asked to complete one of the e-modules and an assignment in cooperation with their trainees. The assignments for the first and fourth meetings were specifically formulated to stimulate interaction between trainer and trainee on EBM. Each of the four modules had its own learning objectives and learning activities. An overview of the educational intervention is presented in Table 1.

**Table 1:** Content of the EBM training

	Day 1: Introduction to EBM	Day 2: EBM – the difference between primary healthcare and second-line medical care	Day 3: How to handle conflicting evidence	Day 4: EBM – the revival
Learning goals	• The GP trainer has knowledge of the different study designs in epidemiologic research.	• The GP trainer can identify the difference between primary healthcare and second-line medical care and its influence on evidence-based practice.	• The GP trainer is aware that reading just one article can lead to false conclusions.	• The GP trainer is aware that the steps of EBM can lead to a relevant answer.
	• The GP trainer can name the advantages and disadvantages of the different study designs.		• The GP trainer can assess the validity, interest and applicability of a systematic review (SR) of side effects.	• The GP trainer can name the success factors of a successful EBM literature search.
	• The GP trainer can name the bias risks of the different designs.			• The GP trainer is motivated to implement this approach in his/her own practice.
Assign-ments (3 h)	• E-learning chapters	• E-learning chapters	• E-learning chapters	• E-learning chapters
	◦ Introduction	◦ Diagnostic research	◦ Therapeutic research	◦ Critical Appraised Topic (CAT)
	◦ Background information	◦ Screening research	◦ Systematic review and meta analyses	• Preparing a mini CAT
	◦ Searching for evidence	• Two learning questions about each of the e-learning chapters	◦ Research on side effects	
	◦ Common statistical constructs	• Search for a sensitivity and specificity of a test used in GP practice.		
	• Composing a PICO			
F2F (3 h)	• E-learning questions	• E-learning questions	• E-learning questions	• E-learning questions
	• Programme	• Programme	• Programme	• Programme
	◦ Learning questions about assignment	◦ Learning questions about assignment	◦ Learning questions about assignment	◦ Learning questions about assignment
	◦ Discussion on value and use of EBM	◦ Discussion about differences between 1^st^- and 2^nd^-line care and EBM: group task	◦ Debate about conflicting evidence	◦ Learning of successes; presentation of each mini CAT
	◦ Exercise: study designs	◦ Influence of prevalence on 1) the outcomes of a diagnostic test, 2) the daily practice of the GP, 3) reasons for unjustified referral to a specialist; discussion and practise with examples	◦ Critical appraisal of a SR	
	◦ Introduction to PICO and literature search			
	◦ Skills training: searching for literature			

This blended course was based on educational evidence on adult learning and evidence regarding technical aspects influencing the educational effectiveness of e-learning. A detailed description of the development of this course has been given elsewhere [26]. In short, based on our learning goals – which require a change of behaviour in practice – and audience (experienced GP trainers), we used the social constructivist learning theory [27] and the adult learning theory [28] as learning principles for the design of our course.

We chose the four-component instructional design (4C/ID) model [29] to give direction to the design of the e-learning component of our programme. This model consists of four interrelated components, namely learning tasks, supportive information, just-in-time (JIT) information and part-task practice. It was developed for designing education or training programmes that continue for a longer period of time and focus on complex learning, that is, the integration of knowledge, skills and attitude and transfer to the work situation.

The GP trainers at the AMC–UvA were randomly divided into 12 groups of 8 to 12 participants. We had four teachers and each teacher guided two to four groups. The GP trainers at the LUMC followed the same course-content but had their own teacher. All teachers were GPs and experienced EBM teachers and received specific training before the course and between the F2F sessions.

### Study design

This cohort study was performed between February 2012 and April 2013. The study design was chosen because of the possibilities to study changes over time. Personal characteristics and competency (knowledge, skills, attitude and intended behavioural change) were assessed using questionnaires at three moments: before the start of the intervention (= T_0_), at the end of the last day of the intervention (= T_1_) and four months after the intervention (= T_2_). After the second and fourth meetings, the participants were asked to describe three intentions for behavioural change, as these are known to be strongly related to actual behavioural change [10].

### Primary outcomes

The first questionnaire sought personal characteristics namely gender, age, year of graduation, experience with research and experience as a trainer. The other questionnaires used are described below and assess 1) EBM knowledge and skills, 2) EBM attitude and 3) EBM behaviour.

1. EBM knowledge and skills
  We used the Fresno test [30, 31] to examine EBM knowledge and skills. This questionnaire has been translated into Dutch and validated for students in allied healthcare [32]. Although the clinical scenarios were adapted for GPs, the same questions and possible answers were used. The questionnaire contains eight open-ended questions with sub-questions, and five multiple-choice questions. Each correctly answered open-ended question generates a maximum of 24 points. The multiple-choice questions are worth between two and four points. The maximum total score is 220 points. The scoring of the open questions of the Fresno test was done in several sessions by three researchers (EtP, MWdW and NvD). During each meeting, scoring was done by one researcher and any ambiguous answers were discussed directly. After all the questionnaires had been scored, a sample of 10 questionnaires from T_0_, T_1_ and T_2_ (a total of 30 questionnaires) were re-examined by another researcher. Scoring of both these questionnaires was compared to assess agreement in scoring.
2. EBM attitude
  Attitude towards EBM was examined using the questionnaire developed by McColl and colleagues for the assessment of EBM attitude in GPs [33], and has been used in multiple studies on EBM [2, 30, 34]. The questionnaire has been forward–backward translated into Dutch [35] and was adjusted, where necessary, for the GP trainers. In the present study, the mean scores on the seven questions assessing the opinion of GP trainers on the attitude towards EBM with the visual analogue scale (VAS) were used to calculate a score on attitude. All questions are scored on a scale of 0 (=very negative) to 100 (=very positive). The scales of negatively formulated questions were inversed for statistical analysis.
3. EBM behaviour
  Behaviour was assessed in two ways. Firstly, six questions on practice behaviour were taken from the McColl questionnaire. These questions focus on EBM behaviour in daily practice and we added the same questions adjusted for their behaviour towards trainees. Also we asked on the practical implementation of the steps of EBM, such as the frequency of formulating a focused search question (a PICO – a question that contains information about Patient details, the Intervention, the Comparison and the Outcome) with the trainee on a practical patient-related question, and the frequency of performing a search and reading an article together.
  Secondly, in the second and fourth meetings, we asked the trainers to name three intended changes in their EBM behaviour in daily practice that the course content up till that moment had provoked (the commitment-to-change method) [10]. The trainers wrote these intentions on cards, which we sent back to them after two months to stimulate actual behavioural change. At T_1_ and T_2_, the trainers were asked to what extent they had fulfilled their intentions, and if they had not been completely fulfilled, the reason for that [36, 37].

### Secondary outcomes

1. To explore the relation between formal assessments as outcome measures and self-reported outcomes, we formulated two self-report questions on EBM knowledge and EBM attitude using a 5-point Likert scale to judge self-rated knowledge and – attitude.
2. To assess whether the characteristics of the trainers had influenced the outcomes of the intervention, we compared groups of trainers on specific characteristics possibly influencing the effectiveness of the training: 1) gender, 2) age (subdivided into 5-year age groups), 3) research experience, 4) starting level of EBM knowledge based (above or below median), 5) EBM teacher and 6) EBM teaching group. We hypothesized that age might have an effect on the EBM competency because of changes in attention for EBM in medical education over the years; that despite the specific training of the courses’ teachers there might be a difference in teaching style that could influence the outcomes; and that although the trainers were randomly assigned, the composition of the training group might influence the outcomes as a result of interactions between the participants.

### Statistical analysis

All data were analysed in SPSS 20.0. An intraclass correlation coefficient (ICC) (two-way mixed model) was calculated to ensure agreement between the examiners of the Fresno test, and was noted as Cronbach’s alpha. Categorical data were summarized as proportions. Continuous data were visually checked for normality. Normally distributed continuous data were expressed as mean with standard deviation (SD), and non-normally distributed data as median and quartiles or as mode. An independent *t*-test was performed to compare the means of continuous, normally distributed data between groups, and a paired-sample *t*-test was performed to compare means at different moments of follow-up. To compare the means of multiple groups with continuous, normally distributed data, an ANOVA was performed with a post hoc Bonferroni test. For the prespecified personal characteristics, variables were divided into groups for comparison. To assess whether the increase in knowledge was greater in participants with the lowest starting level, change from T_0_ to T_1_ was compared between those who scored high and those who scored low at the start (defined by above or below the median score at T_0_) using an independent *t*-test. To assess whether differences between scores were educationally relevant, effect sizes (ES) were calculated using Cohen’s d by dividing the mean difference by the pooled SD*.* A Cohen’s d ≤ .2 was considered small, .5 was considered medium and ≥ .8 was considered large (Cohen)*.*

Spearman’s rank correlation coefficient was used to assess the relation between non-parametric continuous variables. A correlation of .20–.39 was considered weak, .40–.59 as moderate and .60–.79 as strong.

## Results

Because of the small number of participants and the similarity of outcomes at the LUMC, we merged both populations for analysis. Of the 177 GP trainers, 129 (73 %) gave informed consent and filled in the questionnaire at T_0_, 99 (56 %) did so at T_1_ and 89 (50 %) did so at T_2_. Trainers gave no clarification for not participating in the study. The personal characteristics of the included GP trainers are presented in Table 2.

**Table 2:** Personal characteristics of the responding GP trainers in Amsterdam and Leiden

	Academic Medical Center, University of Amsterdam	Leiden University Medical Center
Participants (n)	122	7
Gender (% female)	33.6	28.6
Age (years; mean (SD))	55.9 (5.4)	57.9 (5.0)
Experience as GP (years; mean (SD))	22.4 (7.2)	24.4 (6.7)
GP trainer (years; median (quartiles))	8 (6–11)	10 (7–20)
Research experience (%)	15.6	0

### Primary outcomes

#### Knowledge and skills

The ICC of the Fresno test was high (Cronbach’s α = .96). The mean scores (score 0–220) were T_0_ = 77.6 (SD ±41.0), T_1_ = 120.3 (±33.7) and T_2_ = 96.8 (±38.8) with mean changes on ∆_T1-T0_ = 40.8 (±36.7, *p* < .001), ∆_T2-T0_ = 20.8 (±39.9, *p* < .001) and Δ_T2-T1_ = -17.0 (±35.1, *p* = .001). This results in a proportional 55 % increase in knowledge between T_0_ and T_1_ and of 25 % between T_0_ and T_2_, and a decrease in knowledge of 24 % between T_1_ and T_2_.

#### Attitude

Mean scores per item of the McColl questionnaire are presented in Table 3. The overall mean scores on the VAS items were T_0_: 56.2 (SD ±10.7), T_1_:58.4 (SD ±10.3) and T_2_:57.4 (SD ±9.3) with mean changes on ∆_T1T0_ = 2.2 (SD ±12.8, p = .16), ∆_T2-T0_ = -.87 (SD ±10.0, p = .49) and Δ_T2-T1_ = -.41 (SD ±9.5, p = .76).

**Table 3:** Scores per question of McColl test

	T_0_: Mean (SD)	T_1_: Mean (SD)	T _2:_ Mean (SD)
1. How would you describe your own attitude towards the current promotion of EBM?	63.5 (15.6)	66.0 (15.7)	58.2 (17.0)
2. How would you describe the attitude of most of your colleagues towards EBM?	59.3 (14.0)	52.3 (17.5)	54.3 (14.1)
3. How useful is evidence-based medicine in your day-to-day management of patients?	62.4 (16.8)	63.5 (17.7)	59.8 (18.2)
4. What percentage of your clinical practice is currently evidence based?	57.4 (15.7)	63.0 (14.5)	64.3 (11.1)
5. Practicing EBM improves patient care.	67.0 (17.3)	71.0 (16.9)	70.1 (14.0)
6. EBM is of limited value in general practice because much of primary care lacks a scientific basis.	45.1 (20.6)	56.2 (21.3)	54.8 (20.6)
7. The adoption of EBM, however worthwhile as an ideal, places another demand on already overloaded GPs.	38.1 (21.7)	36.9 (23.1)	40.5 (22.0)
Total score	56.2 (10.7)	58.4 (10.3)	57.4 (9.3)

#### Behaviour

The trainers’ answers to the questions about formulating a PICO, searching for literature and appraising an article with or without their trainee ranged from once a day to once a year. We saw no changes in this behaviour over the time of the intervention.

The five most formulated intentions to change as described after the second and fourth meetings combined were: 1) formulate PICOs, 2) be more critical towards diagnostic tests, 3) calculate the sensitivity and specificity of tests, 4) search in PubMed and make critical appraisal of an article, and 5) pay more attention to EBM in the learning conversations with the trainee. The proportion of GP the trainers who said they fully completed their intentions was 16.7 %, partly completed their intentions 47.6 % and not completed their intentions at all was 35.7 %. The two most frequently mentioned reasons for not fulfilling the intentions were 1) no priority and 2) not having a trainee at the moment. The scores on the questions about fulfilling their intentions showed no differences compared to the questions about fulfilling their intentions to cooperate with their trainees.

### Secondary outcomes

#### Self-rated knowledge and attitude

A weak positive correlation was found between self-rated EBM knowledge and the mean scores on the Fresno test (Table 4). A moderate positive correlation was found between the overall mean score on the McColl test and the self-rated attitude (Table 5).

**Table 4:** Correlation and mean of the total Fresno scores related to self-rated knowledge

Self-rated knowledge	Fresno score					
	T_o_		T_1_		T_2_	
	*r* = .303, p = .001*		*r* = .260, p = .012*		*r* = .33, p = .002*	
	%	Mean (SD)	%	Mean (SD)	%	Mean (SD)
Bad	11.3	71.6 (31.0)	2.2	79.0 (56.6)	10.3	73.7 (36.7)
Moderate	38.3	64.8 (39.5)	30.1	111.9 (35.7)	42.5	86.8 (38.6)
Average	40.9	87.0 (40.9)	53.8	121.6 (30.1)	39.1	109.7 (32.8)
Good	9.6	105.5 (37.9)	14.0	141.1 (34.1)	8.0	115.9 (46.1)
Very good	0	0	0	0	0	0

*Spearman’s rank correlation of the mean score on the Fresno test and self-rated knowledge

**Table 5:** Correlation and mean of the total McColl scores and self-rated attitude

Self-rated attitude	McColl score					
	T_o_		T_1_		T_2_	
	*r* = .52, p < .001*		*r* = .43, p < .001*		*r* = .48, p < .001*	
	%	Mean (SD)	%	Mean (SD)	%	Mean (SD)
Very unimportant	0	0	0	0	0	0
Unimportant	3.5	30.6 (8.0)	1.1	41.0 (N/A)	5.7	47.5 (10.9)
Neutral	33.0	51.0 (8.6)	26.9	52.4 (8.7)	33.0	52.7 (7.3)
Important	68.7	59.9 (8.8)	71.0	60.7 (10.0)	60.2	60.5 (8.3)
Very important	2.6	63.1 (10.0)	1.1	68.8 (N/A)	1.1	77.7 (N/A)

*Spearman’s rank correlation of the mean score on the McColl test and self-rated attitude

#### Influence personal characteristics

The mean scores in knowledge, when comparing trainers with different age, teachers, research experience, and teaching groups, showed no difference at any moment during the study. However, female trainers scored significantly higher than the male trainers on the Fresno test at T_1_ and T_2_ (Table 6). The ES of the Fresno test was high for GP trainers with a low starting level (≤ median T_0_) on both T_1_ and T_2_ and for the high-starting level a high ES was found on T_1_, while almost no effect remained at T_2_ (Table 7).

**Table 6:** Differences in mean Fresno scores between men and women

	Gender	Mean (SD)	p
Fresno score at T_0_	Female	84.2 (37.8)	.253
	Male	75.3 (42.0)	
Fresno score at T_1_	Female	129.4 (27.3)	.040
	Male	114.9 (36.2)	
Fresno score at T_2_	Female	109.1 (37.9sss)	.023
	Male	89.6 (37.9)

**Table 7:** Effect sizes (ES) and mean changes of the Fresno score with low (≤ median) and high (> median) starting level

Fresno score	≤ median		> median	
Time	ES	Mean (SD);∆	ES	Mean (SD);∆
T_1_T_0_	2.67	55.1 (±31.4)	1.06	22.3 (± 27.8)
T_2_T_0_	1.69	39.6 (± 32.6)	0.04	3.9 (± 31.7)
T_2_T_1_	−0.68	−13.5 (± 33.0)	−0.8	−15.2 (± 31.4)

There were no differences in mean scores on the McColl test with regard to gender, age, teacher, teaching groups or research experience.

## Discussion

### Main findings

There was an absolute increase in knowledge after the educational intervention but no change in attitude or behaviour, although the intentions to change behaviour were fulfilled by some trainers. We found a higher increase in knowledge in female GP trainers and trainers with a low starting level, but none of the other characteristics were shown to influence the outcomes. Finally, the scores on the self-report scales on knowledge and attitude and the Fresno test and the McColl test also showed a weak (Fresno test) to moderate (McColl test) positive correlation.

### Comparison to other studies: primary outcomes

We found that a blended learning programme in EBM for GP trainers induces an increase in knowledge.

Four months after the end of the course (T_2_), our respondents showed a 24 % decrease in the mean scores on the Fresno test compared to directly after the course (T_1_) (T_1_ = 120.3 (±33.7); T_2_ = 96.8 (±38.8)), although there was still a 25 % increase compared to knowledge scores at baseline (T_0_ = 77.6 (SD ±41.0); T_2_ = 96.8 (±38.8)). A study by Kok and colleagues [17] among physicians working for the Dutch National Institute of Benefit Schemes started with a higher baseline score on the Fresno test (mean 93.7 (SD 25.9)), which still showed seven months after the intervention an increase (128.2 (SD22.6)) with a slight decrease after 12 months (121.7(25.1)). Literature [38–40] confirms that a response interval (RI) – that is, the time between the last educational intervention and the assessment – negatively influences the retention of knowledge; decay sets in. After a one-year RI, the loss of knowledge gain generally is approximately one third, and within the next year one half [2]. We found after a RI of four months, a loss of knowledge gain of approximately 24 % between T1 and T2, which is comparable to the RI found in other studies [38–40], but not with the study by Kok and colleagues [17]; the loss of knowledge gain was less.

Attitude and behaviour towards EBM show no differences before and after the intervention, although the intention to use EBM more in practice is present and (partly) fulfilled in approximately 50 % of the participants. Earlier studies [41, 42], as the result of a study by Shuval and colleagues [43], suggest a positive influence on attitude and knowledge change after an educational intervention but no actual behaviour change was found. They suggest that an intervention should be both longer (more than three workshops and six out-reach visits) and more extensive, as was done in our study, which however showed similar effects. The theory of reasoned action [10] stated that attitude and subjective norms are predictors of behaviour. Most GP trainers intend to incorporate more EBM behaviour into their daily practice and their role as trainer, but they do not give it any priority. Furthermore, EBM education should be incorporated more fully into the daily routine to minimize the barriers to EBM implementation. Systematic reviews by Ilic and Maloney [22] but also by Coomarasamy and Khan [23] stated that EBM education should integrated theory with ‘real-life’ clinical scenarios’ or should be moved to clinical practice. Although by the practice-based assignments we hoped to integrate EBM more in daily practice, the success of this effort seems limited. Another explanation for the limited rise in attitude in our study, could be that despite our earlier findings on negative attitude in GP trainers [3] our trainers already had a relatively high score on the McColl test at the beginning (all scores were > 50 out of 100; data not reported).

### Comparison to other studies: secondary outcomes

Female trainers scored higher than male trainers on the Fresno test. One explanation could be that the Fresno test is quite linguistic which is said to be an advantage for women, although studies on gender differences and performance or ability, showed inconclusive results on verbal abilities between females and males [44–46]. Also, various studies [47–49] on gender differences in relation to self-regulated learning have been performed, but the results of these studies show inconclusive outcomes as well. Virtanen and Nevgi [49] found a slightly higher score on self-regulated learning by female undergraduate students compared to male students. The differences we found in the mean scores on the Fresno test might have arisen because women are a little better at self-regulated learning compared to men [49] and e-courses requires a great deal of self-regulation. The high effect sizes of the group with a low-starting level is comparable to outcomes of studies in groups receiving no EBM education compared to EBM education which results in a knowledge gain for the intervention groups [50].

Our findings based on the self-report scales on knowledge and attitude versus knowledge/skills and attitude are remarkable. We compared the self-report scales on attitude and knowledge with the scores on the McColl test for attitude and the Fresno test for knowledge/skills. Both show a significant positive correlation (Tables 4, 5 and 6). Several studies [51–53] have found that self-report scales on EBM knowledge are not very reliable or even show a negative correlation. The findings on knowledge cannot be explained by the order of the questions, as the self-report question on knowledge was asked before the questions of the Fresno test. The frame of reference of the respondents therefore cannot have been influenced by the their scores on the Fresno test.

#### Limitations and strengths

An important limitation is our study design; a cohort design. It would have been interesting to compare the learning benefits of this intervention with another educational design. In that case, a RCT design would have been possible. Unfortunately we were not able to use a control group to compare the design of our blended learning intervention with another (blended learning) design. A second limitation is that although the ultimate educational aim is to have GP trainers act as positive role models for GP trainees in EBM behaviour, we did not measure their actual behaviour towards their trainees in daily practice. It is considered very difficult to measure actual EBM behaviour. [54, 55] Existing tools mainly focus on the formulation of PICO and searching for studies, which do not do justice to the more integrative concept of EBM. Within this study, based on the theory of Fishbein, we therefore chose to describe the possible changes in behaviour by expressing intentions. This theory is heavily criticised by Sniehotta, Presseau and Araujo-Soares [56]. Although there are several initiatives to come to an alternative theory, a better explanation of health behaviour change is not yet available. Armitage [57] suggested that the theory of planned behaviour could be one theory within a broader framework and could be used as a benchmark for theories still to come. A third limitation is that this study was performed mostly at one institute in the Netherlands which could have reduced generalizability regarding setting and time.

The strength of this study is that our educational intervention was based on evidence about education especially in blended learning. The group size was another strength: we were able to incorporate 122 GP trainers at the AMC–UvA along with 7 at the LUMC. The validated questionnaires (Fresno and McColl tests) provided a more or less objective assessment of EBM competency.

#### Relevance and future research

Because of the importance of GP trainers as positive role models for their trainees [4, 5], they should be able to incorporate EBM more fully into their daily practice and keep their EBM knowledge and skills more up to date and ready for deployment. EBM behaviour should be visible in the daily practice of the GP trainer. A blended learning educational intervention could be the first step in this process.

In future programs to enhance the transfer of programs on EBM to daily practice: 1) the F2F part should focus even more on assignments performed in clinical practice to assure integration of EBM in daily practice [36, 38]; and 2) EBM should be a component of all annual refresher courses in order to become standard way of thinking for trainers.

Future research could focus on the contribution of the various components of the blended learning program (e-learning, F2F, assignments), on the development of competency-aspects such as attitude, knowledge and behaviour. Based on those studies it might be possible to design schemes addressing those aspects for developing blended learning.

## Conclusion

An intensive blended learning course on EBM for GP trainers induces an increase in knowledge and skills that, although decreased, remains after four months. Attitude and behaviour towards EBM however show no differences before and after the intervention, although GP trainers’ intention to use EBM more often in their practice is present.
